# Supplementary figures and images for: Investigating the direct and indirect effects of forest fragmentation on plant functional diversity
Source: PLoS One. 2020 Jul 2;15(7):e0235210. doi: 10.1371/journal.pone.0235210 (PMC7331995; doi:10.1371/journal.pone.0235210)

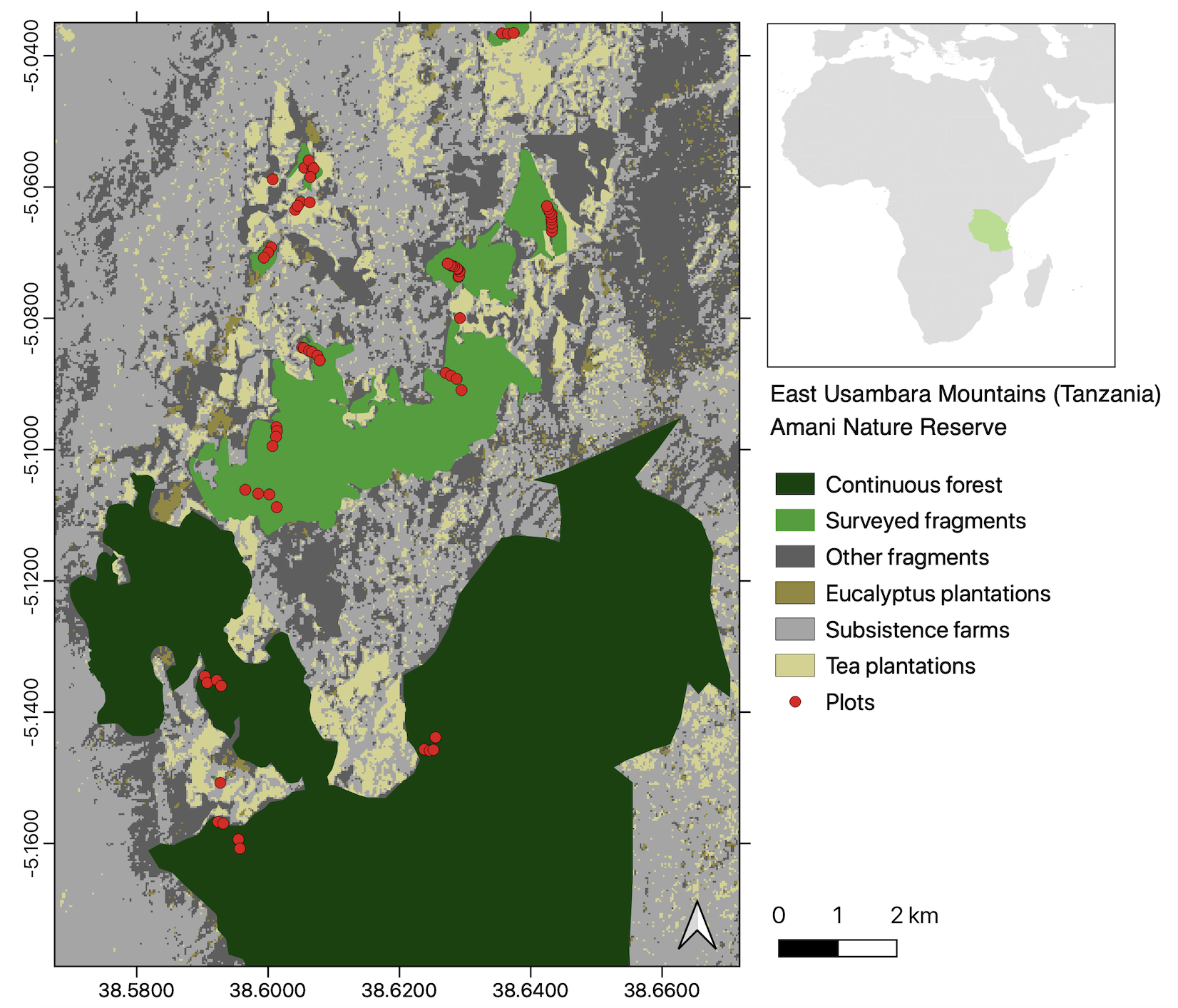

Supplement: S1 Fig — Map of the study area in the East Usambara Mountains of Tanzania. The protected area, Amani Nature Reserve, includes the continuous forest (dark green) and the largest forest fragment (largest fragment in light green). The landcover classification (i.e. tea plantation, Eucalyptus plantation, forest and subsistence farming) was based on a Landsat-8 images from 2016 (courtesy of the U.S. Geological Survey) and performed using the random forest classification extension (r.learn.lm) in GRASS GIS. (TIFF) [file pone.0235210.s001.tiff]
