# Supplementary material for: Investigating the direct and indirect effects of forest fragmentation on plant functional diversity
Source: PLoS One. 2020 Jul 2;15(7):e0235210. doi: 10.1371/journal.pone.0235210 (PMC7331995; doi:10.1371/journal.pone.0235210)
Supplement: S1 Table — (PDF) [file pone.0235210.s002.pdf]

Fragment	Type	Elevation (m)	Area (Km2)	No. of plots
**F12**	Fragment	1131	0.01180	1
**F14**	Fragment	982	0.01279	1
**F6**	Fragment	1024	0.01336	1
**F9**	Fragment	1125.5	0.03967	4
**F13**	Fragment	1124	0.16404	3
**F4**	Fragment	1017.3	0.17016	3
**F7**	Fragment	1065	0.18184	6
**F18**	Fragment	998.3	0.98322	9
**F17**	Fragment	977.6	1.14673	9
**Forest-1**	Large fragment	1046.6	9.51485	18
**Forest-2**	Continuous forest	1043.2	62.0224	12
