# Supplementary material for: Investigating the direct and indirect effects of forest fragmentation on plant functional diversity
Source: PLoS One. 2020 Jul 2;15(7):e0235210. doi: 10.1371/journal.pone.0235210 (PMC7331995; doi:10.1371/journal.pone.0235210)
Supplement: S2 Table — (PDF) [file pone.0235210.s003.pdf]

Species	Family	Dispersal mode[a]	Shade tolerance[b]	Seed Length (mm)	Maximum Height (m)[c]	Wood Density (g/cm^3)[d]
Alangium chinense	Cornaceae	Zoochory	Pioneer	9	24	0.617
Albizia gummifera	Fabaceae	Barochory	Pioneer	10	30	0.544
Alchornea hirtella	Euphorbiaceae	Zoochory	Pioneer	4.5	11	0.413
Allanblackia stuhlmannii	Clusiaceae	Zoochory	shade tolerant	36.8	62	0.646
Allophylus melliodorus	Sapindaceae	Zoochory	shade tolerant	4	20	0.513
Alsodeiopsis schumannii	Icacinaceae	Zoochory	shade tolerant	23.7	16	0.577
Anisophyllea obtusifolia	Anisophylleaceae	Zoochory	shade tolerant	21.4	50	0.739
Annickia kummeriae	Annonaceae	Zoochory	shade tolerant	21.7	22	0.447
Anthocleista grandiflora	Gentianaceae	Zoochory	Pioneer	1	50	0.532
Antiaris toxicaria	Moraceae	Zoochory	Pioneer	12.5	40	0.383
Aoranthe penduliflora	Rubiaceae	Zoochory	Pioneer	1.3	18	0.807
Beilschmedia kweo	Lauraceae	Zoochory	shade tolerant	50.8	40	0.574
Bersama abyssinica	Melianthaceae	Zoochory	non pioneer light demanding	11	30	0.618
Blighia unijugata	Sapindaceae	Zoochory	shade tolerant	10.5	18	0.710
Bridelia micrantha	Phyllanthaceae	Zoochory	Pioneer	8.1	20	0.553
Camellia sinensis	Theaceae	Zoochory	NA	14.1	2.5	0.550
Celtis africana	Cannabaceae	Zoochory	non pioneer light demanding	7	50	0.594
Celtis gomphophylla	Cannabaceae	Zoochory	non pioneer light demanding	6.4	35	0.594
Celtis mildbraedii	Cannabaceae	Zoochory	non pioneer light demanding	6	35	0.594
Cephalosphaera usambarensis	Myristicaceae	Zoochory	shade tolerant	49.1	57	0.430
Chrysophyllum perpulchrum	Sapotaceae	Zoochory	shade tolerant	18.9	52	0.655
Cleistanthus polystachyus	Phyllanthaceae	Zoochory	shade tolerant	6	30	0.633
Cola greenwayi	Malvaceae	Zoochory	shade tolerant	14.5	35	0.601
Cola usambarensis	Malvaceae	Zoochory	shade tolerant	15	8	0.601
Cordia sp	Cordiaceae	Zoochory	Pioneer	8	15	0.510
Craibia zimmermannii	Fabaceae	Barochory	shade tolerant	27	28	0.620
Cylicomorpha parviflora	Caricaceae	Zoochory	Pioneer	7	22	0.226
Cynometra brachyrrhachis	Fabaceae	Barochory	shade tolerant	20.4	50	0.803
Cynometra longipedicellata	Fabaceae	Barochory	shade tolerant	21	42	0.803
Cynometra sp.	Fabaceae	Barochory	shade tolerant	20	35	0.803
Dasylepis integra	Achariaceae	Zoochory	shade bearing	8.5	12	NA
Drypetes gerrardii	Putranjivaceae	Zoochory	shade tolerant	12.3	50	0.707
Drypetes usambarica	Putranjivaceae	Zoochory	shade tolerant	15	45	0.707
Englerodendron usambarense	Fabaceae	Barochory	shade tolerant	40	35	0.678
Entandrophragma excelsum	Meliaceae	Anemochory	non pioneer light demanding	40	35	0.536
Erythrococca fischeri	Euphorbiaceae	NA	NA	3.5	7.5	NA
Ficus exasperata	Moraceae	Zoochory	Pioneer	0.5	30	0.412
Ficus sur	Moraceae	Zoochory	Pioneer	0.01	23	0.412
Funtumia africana	Apocynaceae	Anemochory	Pioneer	2	28	0.424
Greenwayodendron suaveolens	Annonaceae	Zoochory	shade tolerant	10	32	0.564

Heinsenia diervilleoides	Rubiaceae	Zoochory	shade tolerant	9.7	28	0.636
Ilex mitis	Aquifoliaceae	Zoochory	shade tolerant	3.9	40	0.558
Isoberlinia scheffleri	Fabaceae	Barochory	shade tolerant	34.5	60	0.688
Isolona heinsenii	Annonaceae	Zoochory	shade tolerant	12.2	15	0.564
Lagynias parviflora	Rubiaceae	Zoochory	shade tolerant	12	20	0.636
Leptonychia usambarensis	Malvaceae	Zoochory	non pioneer light demanding	12	22	0.476
Macaranga capensis	Euphorbiaceae	Zoochory	Pioneer	4	35	0.381
Maesa lanceolata	Primulaceae	Zoochory	Pioneer	0.5	11	0.676
Maesopsis eminii	Rhamnaceae	Zoochory	Pioneer	20	40	0.384
Magnistipula butayei	Chrysobalanaceae	Zoochory	shade tolerant	40	25	0.781
Maranthes goetzeniana	Chrysobalanaceae	Zoochory	shade tolerant	30	52	0.817
Margaritaria discoidea	Phyllanthaceae	Zoochory	non pioneer light demanding	5	20	0.664
Mesogyne insignis	Moraceae	Zoochory	shade tolerant	13.2	15	0.541
Milicia excelsa	Moraceae	Zoochory	non pioneer light demanding	2	50	0.569
Milletia dura	Fabaceae	Barochory	Pioneer	10	18	0.678
Morinda asteroscepa	Rubiaceae	Zoochory	Pioneer	3	30	0.558
Myrianthus holstii	Urticaceae	Zoochory	non pioneer light demanding	12.3	30	0.450
Newtonia buchananii	Fabaceae	Anemochory	shade tolerant	57.5	57	0.574
Ocotea usambarensis	Lauraceae	Zoochory	shade tolerant	3	45	0.523
Oxyanthus speciosus	Rubiaceae	Zoochory	shade bearing	27	18	NA
Parinari excelsa	Chrysobalanaceae	Zoochory	non pioneer light demanding	36.8	50	0.702
Phoenix reclinata	Arecaceae	Zoochory	non pioneer light demanding	12.5	20	0.557
Placodiscus amaniensis	Sapindaceae	Zoochory	shade tolerant	8	29	0.793
Pleiocarpa pycnantha	Apocynaceae	Zoochory	non pioneer light demanding	9.5	30	0.567
Polyceratocarpus scheffleri	Annonaceae	Zoochory	shade tolerant	17	16	0.564
Polyscias fulva	Araliaceae	Zoochory	Pioneer	4	45	0.373
Pouteria adolfi-friedericii	Sapotaceae	Zoochory	shade tolerant	32.7	50	0.714
Pterocarpus tinctorius	Fabaceae	Anemochory	non pioneer light demanding	20	48	0.587
Quassia undulata	Simaroubaceae	Zoochory	non pioneer light demanding	35.8	50	0.324
Rauvolfia caffra	Apocynaceae	Zoochory	Pioneer	10	40	0.475
Rawsonia lucida	Achariaceae	Zoochory	shade tolerant	8	18	0.745
Rytiginia flavida	Rubiaceae	Zoochory	shade tolerant	7	5	0.636
Rytigynia sp.	Rubiaceae	Zoochory	shade tolerant	7	12	0.636
Rytigynia xanthhotricha	Rubiaceae	Zoochory	shade tolerant	7	15	0.636
Schefflerodendron usambarense	Fabaceae	Barochory	shade tolerant	16.9	18	0.678
Shirakiopsis elliptica	Euphorbiaceae	Zoochory	non pioneer light demanding	5.3	30	0.414
Sorindeia madagascariensis	Anacardiaceae	Zoochory	shade tolerant	21.8	30	0.560
Strombosia scheffleri	Strombosiaceae	Zoochory	shade tolerant	18.3	40	0.747
Symphonia globulifera	Clusiaceae	Zoochory	shade tolerant	17.5	20	0.618
Synsepalum cerasiferum	Sapotaceae	Zoochory	shade tolerant	16.9	50	0.678
Synsepalum msolo	Sapotaceae	Zoochory	shade tolerant	20.3	50	0.678
Syzygium guineense	Myrtaceae	Zoochory	shade tolerant	7	40	0.649
Tabernaemontana pachysiphon	Apocynaceae	Zoochory	Pioneer	12.5	18	0.525

Tabernaemontana stapfiana	Apocynaceae	Zoochory	Pioneer	20	13	0.525
Tricalysia pallens	Rubiaceae	Zoochory	shade tolerant	4	40	0.100
Trichilia dregeana	Meliaceae	Zoochory	shade tolerant	16.6	35	0.634
Trichilia emetica	Meliaceae	Zoochory	shade tolerant	17.5	30	0.634
Trilepisium madagascariense	Moraceae	Zoochory	shade tolerant	14.3	30	0.541
Turraea holstii	Meliaceae	Zoochory	shade tolerant	4.5	18	0.663
Uvariodendron usambarense	Annonaceae	Zoochory	shade tolerant	21.4	15	0.564
Vangueria infuasta	Rubiaceae	Zoochory	shade tolerant	25	7	0.657
Vepris nobilis	Rutaceae	Zoochory	shade tolerant	5.5	25	0.659
Vitex amaniensis	Lamiaceae	Zoochory	shade tolerant	13	30	0.549
Xylopia aethiopica	Annonaceae	Zoochory	Pioneer	6	45	0.678
Xymalos monospora	Monimiaceae	Zoochory	shade tolerant	11.8	17	0.588
Zanha golungensis	Sapindaceae	Zoochory	shade tolerant	21.7	24	0.795
Zanthoxylum usambarense	Rutaceae	Zoochory	Pioneer	6	30	0.610
Zanthoxylum gilletti	Rutaceae	Zoochory	Pioneer	3.5	35	0.610
Zenkerella grotei	Leguminosae	Barochory	shade bearing	15	22	NA

[a] dispersal mode: zoochory (animal-dispersal); anemochory (wind-dispersal); and barachory (gravity or explosive dispersal); data from Chapman et al. (2016), the African Tree Database (https://figshare.com/articles/Plant_animal_interactions_from_Africa/1526128) and N.J. Cordeiro & H.J. Ndangalasi, unpublished data.
[b] shade tolerance: pio = pioneer; shade tol = shade tolerance; non-pio light dem = non-pioneer light demanding; data classifications from Ouédraogo et al. (2013, 2016), supplemented information from Ruffo et al. (1989) and Lovett et al. (2006).
[c] tree height: sourced from Lovett et al. (2006) and http://www.prota.org/
[d] wood density (g/cm^3) sourced from global wood density database (Chave *et al.* 2009; Zanne *et al.* 2009).

LITERATURE CITED

Chave, J., Coomes, D., Jansen, S., Lewis, S.L., Swenson, N.G. & Zanne, A.E. (2009) Towards a worldwide wood economics spectrum. *Ecology Letters*, **12**, 351–366.

Lovett, J., Sørensen, L. & Lovett, J. (2006) *Field Guide to the Moist Forest Trees of Tanzania*. Society for Environmental Exploration.

Ruffo, C.K., Mmari, C., Kibuwa, S.P., Lovett, J., Iversen, S., Hamilton, A.C. & IUCN Tropical Forest Programme. (1989) A Preliminary List of Plant Species Recorded from the East Usambara Forests. *Forest conservation in the East Usambara Mountains, Tanzania* (eds A.C. Hamilton & R. Bensted-Smith), pp. 157–180. IUCN, Gland, Switzerland.

Zanne, A.E., Lopez-Gonzalez, G., Coomes, D.A.A., Ilic, J., Jansen, S., Lewis, S.L.S.L., Miller, R.B.B., Swenson, N.G.G., Wiemann, M.C.C. & Chave, J. (2009) Global wood density database. *Dryad*, **235**, 33.
