# Supplementary material for: Investigating the direct and indirect effects of forest fragmentation on plant functional diversity
Source: PLoS One. 2020 Jul 2;15(7):e0235210. doi: 10.1371/journal.pone.0235210 (PMC7331995; doi:10.1371/journal.pone.0235210)
Supplement: Attachment — Submitted filename: Responses to reviewers.docx [file pone.0235210.s004.docx]

April 5, 2020

Dear Dr. Heinze,

We thank you for handling our original submission of our manuscript entitled “Investigating the direct and indirect effects of forest fragmentation on plant functional diversity” for consideration as a *Research Paper* in PlosOne as part of the Biodiversity Conservation Collection. Enclosed you will find a revised version of the manuscript that has been approved by my coauthors. We appreciate your consideration on the revised version.

In the following, we respond to each individual comment one-by-one. We hope you find the revisions satisfactory.

Sincerely,
Jenny Zambrano (on behalf of my co-authors)

---------------------------------------------------------------------------------------------------------------------

Additional Editor Comments (if provided):

After some struggle (19 potential reviewers were contacted), we finally obtained three high quality reviews that help a lot in shaping this manuscript further into a very interesting contribution. The three reviews are divided in their recommendation, maybe because they go to different levels of depth, but for me the long and short of it is that some aspects require additional thoughts (or analyses), some of the wording needs careful reconsideration, and some technical aspects need attention (e.g. in some figures). I would like to invite the authors to take care of all issues mentioned by the reviewers and provide comment/action for all of these.

Journal Requirements:

When submitting your revision, we need you to address these additional requirements:

1. Please ensure that your manuscript meets PLOS ONE's style requirements, including those for file naming. The PLOS ONE style templates can be found at <http://www.plosone.org/attachments/PLOSOne_formatting_sample_main_body.pdf> and <http://www.plosone.org/attachments/PLOSOne_formatting_sample_title_authors_affiliations.pdf>

**Authors: Done**

2. In your Methods section, please provide additional location information of the study area, including geographic coordinates for the data set if available.

**Authors: We have included the main latitude and longitude coordinate in the main text and all GIS coordinates in the online database available at** <https://knb.ecoinformatics.org/view/doi:10.5063/F1KS6PX9>

3. We note that you have stated that you will provide repository information for your data at acceptance. Should your manuscript be accepted for publication, we will hold it until you provide the relevant accession numbers or DOIs necessary to access your data. If you wish to make changes to your Data Availability statement, please describe these changes in your cover letter and we will update your Data Availability statement to reflect the information you provide.

**Authors: The data is available at:** <https://knb.ecoinformatics.org/view/doi:10.5063/F1KS6PX9>

4. We note that Figure S1 in your submission contain map images which may be copyrighted. All PLOS content is published under the Creative Commons Attribution License (CC BY 4.0), which means that the manuscript, images, and Supporting Information files will be freely available online, and any third party is permitted to access, download, copy, distribute, and use these materials in any way, even commercially, with proper attribution. For these reasons, we cannot publish previously copyrighted maps or satellite images created using proprietary data, such as Google software (Google Maps, Street View, and Earth). For more information, see our copyright guidelines: <http://journals.plos.org/plosone/s/licenses-and-copyright>.

**Authors: We have now use Landsat-8 images from 2016, courtesy of the U.S. Geological Survey.**

We require you to either (a) present written permission from the copyright holder to publish these figures specifically under the CC BY 4.0 license, or (b) remove the figures from your submission:

a.    You may seek permission from the original copyright holder of Figure S1 to publish the content specifically under the CC BY 4.0 license.

We recommend that you contact the original copyright holder with the Content Permission Form (<http://journals.plos.org/plosone/s/file?id=7c09/content-permission-form.pdf>) and the following text:

“I request permission for the open-access journal PLOS ONE to publish XXX under the Creative Commons Attribution License (CCAL) CC BY 4.0 (<http://creativecommons.org/licenses/by/4.0/>). Please be aware that this license allows unrestricted use and distribution, even commercially, by third parties. Please reply and provide explicit written permission to publish XXX under a CC BY license and complete the attached form.”

Please upload the completed Content Permission Form or other proof of granted permissions as an "Other" file with your submission.

In the figure caption of the copyrighted figure, please include the following text: “Reprinted from [ref] under a CC BY license, with permission from [name of publisher], original copyright [original copyright year].”

b.    If you are unable to obtain permission from the original copyright holder to publish these figures under the CC BY 4.0 license or if the copyright holder’s requirements are incompatible with the CC BY 4.0 license, please either i) remove the figure or ii) supply a replacement figure that complies with the CC BY 4.0 license. Please check copyright information on all replacement figures and update the figure caption with source information. If applicable, please specify in the figure caption text when a figure is similar but not identical to the original image and is therefore for illustrative purposes only.

The following resources for replacing copyrighted map figures may be helpful:

USGS National Map Viewer (public domain): <http://viewer.nationalmap.gov/viewer/>

The Gateway to Astronaut Photography of Earth (public domain): <http://eol.jsc.nasa.gov/sseop/clickmap/>

Maps at the CIA (public domain): <https://www.cia.gov/library/publications/the-world-factbook/index.html> and <https://www.cia.gov/library/publications/cia-maps-publications/index.html>

NASA Earth Observatory (public domain): <http://earthobservatory.nasa.gov/>

Landsat: <http://landsat.visibleearth.nasa.gov/>

USGS EROS (Earth Resources Observatory and Science (EROS) Center) (public domain): [http://eros.usgs.gov/#](http://eros.usgs.gov/)

Natural Earth (public domain): <http://www.naturalearthdata.com/>

5.Please include captions for your Supporting Information files at the end of your manuscript, and update any in-text citations to match accordingly. Please see our Supporting Information guidelines for more information:[http://journals.plos.org/plosone/s/supporting-information](https://urldefense.proofpoint.com/v2/url?u=http-3A__journals.plos.org_plosone_s_supporting-2Dinformation&d=DwMGaQ&c=C3yme8gMkxg_ihJNXS06ZyWk4EJm8LdrrvxQb-Je7sw&r=venneP8rhI6q9hFApU1ni00etyCBixq84sV-1EbxK7g&m=FClV3CQAGcDJtyV6AAHrJgelAV2eUBodjls1r_CqALs&s=dgYN9IoB8Lc01D1Bl1wrvjvzXtl58DjqHvhoBTl_9js&e=)

­ **Authors: Done** ________________________________________________________________________________________________________

Reviewers' comments:

Reviewer's Responses to Questions

**Comments to the Author**

**Reviewer #1**:

Zambrano et al. present an interesting work using a multi-scale SEM approach to tease apart the relative importance of suits of fragmentation effects on functional diversity in forest ecosystems. By exploring five key functional traits (i.e., seed length, dispersal mode, shade tolerance, maximum tree height and wood density), they found forest fragmentation directly and indirectly influences tree functional richness and evenness. Fragment area combing with edge habitat and shape complexity reduce functional richness and evenness for those traits related to resource acquisition as well as selecting fast grow tree species. Overall, I enjoy reading the work. The manuscript is well organized, and the framework, results and discussion are clear. This work is also timely important for forest management. I have only one major concern about quantifying functional diversity.

**Authors: Thank you for your support.**

To account for unequal sampling effort, functional diversity indices were calculated through rarefied communities. It’s unclear for me about the rationale to use the mean 1000 random draws to represent the functional diversity indices. I am wondering whether the results for the expected functional indices are the same as the observed one. In addition, I cannot follow the rationale in Lines 230-234, too.

**Authors: We removed the analysis using 1000 random draws of the rarefied community because all plants were exhaustively sampled in each plot. We used Community Weighted Means as a complement to functional diversity indices to better understand the functional shift observed in the studied area.**

Minor concerns:
Line 112: What does UNESCO represent?

**Authors: We have written out UNESCO so all readers are aware of what it stands for.**

Line 248: Do you mean AICcs are less than 2?

**Authors: We have deleted this information since we used a full model and no model selection was required.**

Lines 299-307: This section can be moved to Methods section.

**Authors: Thank you for the suggestion; we moved part of this section to metods and left the results of this analysis to clarify our observations about distance to edge and, that complements the previous section.**

Line 476: Full journal name?

**Authors: Done**

**Reviewer #2:**

GENERAL COMMENTS
A sound study that is well written with short-comings duly acknowledged by authors. My main comment focusses on the need for a greater consideration of animal vectors given that the majority of trees were animal dispersed (e.g. rather than wind dispersed). There was no mention on the habitat requirements/preferences on the animal vectors that would no doubt have an impact on plant colonisation (e.g. edge specialists vs core habitat requirements for fauna). More comments on this point provided below.

**Authors:** **Thank you for your suggestions. The concerns raised about animal seed dispersal are valid given that we did not clarify fully that we are evaluating functional diversity of adult tree communities post-fragmentation. We censused all trees ≥ 10 cm DBH. The mature trees sampled in our census are largely remnants from pre-fragmentation, and for the most part, pioneer trees sampled in our census would have colonized post-fragmentation.  Had we analysed functional diversity of early life history stages (i.e., seed, seedling, and saplings/recruits), we would have been able to make inferences about seed dispersal.**

ABSTRACT
L25, ‘fragment … scale’, I assume you mean ‘local … scale’? (e.g. as referred to in L109)
**Authors: We replaced “fragment” by “local”**

INTRODUCTION
The introduction lacks any material on the role of animals in seed dispersal and plant colonisation (of both edges and forest interior). Given that there are very few wind-dispersed species in the forest and that nearly all are animal dispersed (including most of the pioneer species, from Table S1), there needs to be some consideration of fragmentation effects on colonisation by animal dispersed species – and I would also think some associated hypotheses?

**Authors: Thank you for your suggestions. However, as we previously mentioned we have analyzed mostly pre-fragmentation tree communities. Pioneers would be the main guild that have colonized post-fragmentation, and we have made this clearer throughout the Mss.**

L52, L58, missing space

**Authors: Corrected**

L67, clarify what you mean by a ‘harsh matrix’

**Authors: We mean a low quality matrix. We have included this clarification line 63.**

L78, still not clear what you mean by ‘fragment-scale’ (given fragment size can vary by orders of magnitude) – clarify so that this is clear in its subsequent use throughout

**Authors: We have clarified this in line 74.**

L93, passive sentence structure – revise

**Authors: Done**

L114-125, there are many predictions included here – would it be possible to tighten up this paragraph? E.g. L115, 116, 123 each relate to pioneer species and their preference for edges.

**Authors: We have completely rewritten the hypotheses to distinguish fragmentation effects on traits related to acquisition and traits associated with dispersal. Thank you for your suggestion.**

L116, what about potential for long-distance dispersal via animal vectors?

**Authors: As we previously mentioned we have analyzed mostly pre-fragmentation tree communities. In order, to determine the effects of long-distance dispersal we need to study seedling communities which was not the scope in this study.**

L127, ‘fragment-scale fragmentation’ – meaning? (also L128)

**Authors: We have removed “fragmentation”**

MATERIALS AND METHODS
L144-148, for an international audience, it would be useful to include associated plant families (for those not familiar with the species)

**Authors: Thank you for the suggestion. We have included the associated plant families.**

L147, closing bracket here but there is no opening bracket

**Authors: We have removed the closing bracket**

L157, 55 plots does not match the total number of plots shown in FigS1, nor the number of plots listed in Table S1.

**Authors: Corrected**

L158, 20 × 20 m (not 20x20m)

**Authors: Corrected**

L159, some fragments seem to have many more plots (Fig S1)?

**Authors: That’s correct. We already made this apparent in the text.**

L161, define DBH, measured for what? Presumable DBH and height only? How was height measured? Some indication of level of accuracy is needed.

**Authors: Corrected. In addition, we have removed “height” since it was not included in any of the analyses.**

L199-200, does this mean the metric considered the proportion of edge in contact with each of the different types of cultivated land? (e.g. given that each would potentially represent different habitat value for dispersal vectors, pollinators etc., differential effects on microclimate …)

**Authors: Here we mean matrix quality, and we have clarified in the methods (lines 209-217) how we define and calculate matrix quality based on the different types of cultivated land surrounding fragments.**

RESULTS
Figure 3, from the scale shown on the map (Fig S1), I don’t know how distance to the continuous forest could be in the order of 1000’s of km (the scale shown in Fig S1 may also be incorrect).
**Authors: Corrected**

DISCUSSION
L339-345, yes!

**Authors: Thank you.**

L367, avoid starting paragraph with ‘Furthermore’

**Authors: Revised.**

L373, ‘compared’ not ‘compare’

**Authors: Corrected.**

L398, I don’t quite follow – wouldn’t it be easier to pick up a trend is you have a large range of seed dispersal modes? Moreover, the majority of the species were zoochorous – so perhaps it is the lack of diversity in dispersal modes that prevented detection of any trends? Zoochorous species may be ant dispersed, dispersed via ingestion or dispersed via adhesion – perhaps considering the finer levels of these dispersal modes would help given the flow on implications to dispersal distances and also habitat requirements of the animal vectors. [I see you make reference to this point in L405-407; however outside of gape-width, could you make finer groupings based on the type of animal vector – or are they all bird dispersed via ingestion?]

**Authors: Thank you. This is a good point. Unfortunately, we do not have data on the finer-scale categories of dispersal mode. We have altered this section as the new results include an effect of fragmentation on anemochory discussed in lines 401-406, and in addition, we clarify earlier that we are analyzing post-fragmentation communities of mature trees, much of which are remnants of pre-fragmentation, and hence changes in the diversity of dispersal modes are driven by pioneer species.**

CONCLUSION
L421-428, this is introductory material.

**Authors: We have deleted part of this section and move part of it to the end of the conclusion to reflect the important of using an approach such as the one we use in this study to better understand fragmentation effects and apply it to management efforts.**

SUPPLEMENTARY
Multiple font types used, inconsistent number of decimal places used -this needs tightening up.

**Authors: Corrected.**

Figure S1, Latitude and Longitude should be included along the edges of the map border. It looks like the scale legends are incorrect (possibly in both maps). What does the ‘white’ area represent? If the area between the forest fragments supports other land uses (e.g. tea plantations as indicated in text), then this should be shown.

**Authors: Corrected.**

Table S1, the number of plots listed in the table does not seem to match the plots shown in Figure S1? I wouldn’t describe this table as one of forest metrics as the only metric shown in Fragment Area.

**Authors: Corrected.**

Do you need to differentiate the sizes according to small, medium, large (categorical attributes) given you have the actual area?

**Author: We have deleted this column.**

Is the large green fragment considered one fragment? The numbers of fragments listed in the table don’t seem to match Figure S1.

**Authors: Corrected.**

Table S2, units are needed for Seed length, Height, Wood density.

**Authors: Corrected.**

**Reviewer #3:** In this manuscript Zambrano et al analyze the response of different functional diversity metrics to fragmentation variables separated into landscape and fragment-level using SEM. They report various responses of functional richness, evenness and divergence to fragmentation.

**Authors: Thank you for your support.**

The paper is interesting and generally well-written, my main suggestions for improvments are:

- check the consistency between the reported results and the statements in the discussion, for instance in the result a decrease in functional richness with distance to the edge is reported while in the discussion the opposite is stated

**Authors: Done.**

- the result section could be improved by separating the discussion of the inter-relation between the fragmentation variables from the discussion of the effect of fragmentation on functional diversity

**Authors: Thank you for the suggestion. We have divided the results from the SEMs into two sections: 1) the inter-relation between fragment variables and 2) effects on functional diversity.**

Additionaly I have the following more detailed comments:

Line 37: this manuscript is about functional diversity, I recomend to change „community composition“ by „functional diversity“ or similar

**Authors: Corrected.**

Line 52: Replace „their local diversity“ by „local functional diversity“

**Authors: Done**

Line 58: Unclear here what multivariate / univariate refer to, maybe drop it?

**Authors: Done.**

Line 74-89: Nice paragraph.

**Authors: Thank you!**

Line 95: One verb to much, choose one between identify and understanding.

**Authors: Corrected.**

Line 96-97: The interesting properties of SEM in this context could be made a bit clearer here. Something like: „may miss critical indirect effects between fragment-level and landscape-level fragmentation variables“

**Authors: Corrected.**

Line 107: I would be more specific here, the introduction focused on fragmentation only, „drivers“ is a bit vague here in that regard. Would suggest to replace with „fragmentation effects“.

**Authors: Corrected.**

Line 114-131: The hypothesis would need some greater consistency in their generality. For instance the first part „forest fragmentation is decreasing functional diversity“, is very vague given the objectives of the manuscript to disentagle direct and indirect relation across multiple scales. In this hypothesis, which aspect of forest fragmentation are you talking about? Which aspect of functional diversity? Similarly, the text under the (ii) subheaders is also very vague. I would recommend to only keep the three detailed points (matrix quality / isolation, fragment size and edge effects) separated into (i) to (iii).

**Authors: We have included the information of how functional diversity was defined and the expected results on diversity of traits related to resource acquisition and dispersal, as well as, the expected interactions between fragment- and landscape-level processes.**

Line 136: What range?
**Authors: Clarified.**

Line 138: Precipitation is usually given in mm

**Authors: Corrected.**

Line 214: remove „that describe“ and add a colon :

**Authors: Done**

Line 222: How was abundance derived? Number of stems? DBH? Crown cover?

**Authors: Number of stems. We have included this information.**

Line 226: This require clarification because above it is stated that all trees with >1m DBH were identified to species.

**Authors: Corrected**

Line 244: Stepwise selection via AICc is an exploratory procedure that does not seem to fit to the confirmatory framework of the manuscript (clear hypothesis with predictions are set in the introduction). Why not just use the full model?

**Authors: Thank you for the recommendation. We now present and discuss the results from full models.**

Line 260: I find the results as they stand now rather confusing because (at least) two different aspects are discussed at the same time: (i) the relation between the fragmentation variables (potential indirect effects) and (ii) the response of functional diversity to the fragmentation variables: I would recommend to separate the results into different subsections, in a first one the relations between the fragmentation variables would be discussed, and in subsequent ones the response of the different functional diversity metrics. Dropping the stepwise approach would also make the message clearer because now the inter-relation between the fragmentation variables is changing between the different functional diversity metrics which is a bit counter-intuitive. Working with a well thought-off full model would prevent this dispersion.

**Authors: Thank you for the recommendations. We have now divided the results into two sections and drop the stepwise approach.**

Line 285: „functional divergence tended to decrease“, with a p-value of 0.6, I find this a bit cheeky to give a direction to this effect that clearly could go in any (or no) direction.

**Authors: Following reviewer suggestions on analysis, we now report new results pertaining to functional divergence that show a strong relationship with shape complexity.**

Line 289: Please also report here the slopes and their p-value to be consistant with previous sections

**Authors: Done.**

Line 327: This is in contradiction with results reported line 266 for functional richness and in the result section I can see no reference to edge effects on functional eveness. Please check once again your result and derive the relevant conclusions from it!
**Authors: We have corrected this.**

Line 343-345: Good point! Maybe it is an option to already start looking at beta and gamma functional diversity in this manuscript?

**Authors: Thank you for your recommendation. This is something we tried in a previous analysis; however, due to the low replication at the landscape level and variation in sampling effort we were not able to draw strong conclusions from this analysis.**

Line 351-352: Again not supported by your results …
**Authors: Corrected.**

Table 1: Some of the coefficient estimates are very tiny (1e-20), why is that? What is the unit of your response variables? Did you consider re-scaling your response to improve model estimation (underflowing issue might arise with such tiny values)?

**Authors: Thank you for your suggestion. We have rescaled the CWM values.**

Figure 1: This figure is a bit confusing because in the SEM literature using a box (functional diversity) with arrows to different variables (functional richness …) is traditionally used to represent a latent variable. This is not the case here so to prevent confusion I would recommend to have one box with functional diversity and below it in bracket functional richness … Please also add in the legend that the different functional diversity metric were fitted in separate models.

**Authors: Corrected.**

Figure 2: Where is functional divergence? Also difference between significant / non-significant paths is not clear at all. Consider using a color scale. Also I am not sure that using line types (dashed, not dashed) to differentiate between direct and indirect effects is helpful here.

**Authors: Corrected and we have included a figure including functional divergence.**


Figure S1: Some plots seem to be very close together, did you consider trying a Mantel test on the model residuals to check if there are any spatial autocorrelation present?

**Authors:** **Thank you for the suggestion; however, we believe that** **the measurements of fragment and landscape properties (i.e. distance of plot to edge of fragment, distance of fragment to continuous forest), included in this study, take into count the spatial context of the focal units. We do include information on distance of plot to fragment edge and distance of fragment to forest, so we would expect this would take into account some of the spatial signature.** **Of course, we are not accounting for variation in variables such soil and topography, to give just two examples, hence we cannot be completely certain of the spatial signature stems from the spatial fragmentation processes or the underlying topography/soil.**
