# Supplementary material for: Investigating the direct and indirect effects of forest fragmentation on plant functional diversity
Source: PLoS One. 2020 Jul 2;15(7):e0235210. doi: 10.1371/journal.pone.0235210 (PMC7331995; doi:10.1371/journal.pone.0235210)
Supplement: Attachment — Submitted filename: PlosONE_response_to_reviewers.docx [file pone.0235210.s005.docx]

Dear Dr. Heinze,

We thank you for your handling of our previous submission to PlosONE entitled: Investigating the direct and indirect effects of forest fragmentation on plant functional diversity (ID: PONE-D-19-3430R1). The manuscript received two detailed expert reviews. There were a few remaining points that needed to be addressed leading the decision of minor revision.

Detailed responses can be found below. The suggestions made by the reviewers greatly helped to improve the work and the manuscript.

We thank you again for your consideration and we hope you will find this new version of the manuscript suitable for publication in PlosONE.

Sincerely,

Jenny Zambrano

Additional Editor Comments (if provided):

All reviewers are impressed by the additional work done for improving the manuscript. It is considered (almost) ready for publication. I think the few remaining points can easily be addressed (some may just be a matter of re-wording in order to gain clarity in expression); e.g. the hypotheses are mentioned by two of the reviewers. Also carefully consider the other comments please.

**Authors: Thank you for handling the paper.**

Reviewer #1: I've read the revised manuscript. The authors have done an excellent work. All of my concerns have been addressed. Congratulations!

**Authors: Thank you for your support and for your previous suggestions.**

Reviewer #2: GENERAL
A much improved manuscript. The authors have diligently addressed the numerous reviewer comments. I only have a few minor comments.
My comments relate to the unannotated version.

**Authors: Thank you for your support and for your previous suggestions.**

INTRODUCTION
L65, Are you suggesting that animal-dispersed species are highly dispersed due to their smaller seed? Please clarify – many animal-dispersed species have large seed and are dispersed long distances by being carried by animals (either internally / externally). L120 mentions abiotically-dispersed species with smaller seeds – to I suspect the sentence in L65 just needs to be rephrased for clarity.

**Authors: Corrected**

L109-113, the hypothesis is poorly expressed – please clarify. I assume you are expecting a decline in functional richness, evenness and divergence?

**Authors: Corrected**

MATERIALS AND METHODS
L255, space needed (100 m, not 100m)

**Authors: Corrected**

RESULTS
L303-317, given the results are provided in Table 1, I see no need to list estimate and se in the text (the table could also include t and p).

**Authors: Corrected**

L324-332, I could not find Figure 3 in the revised version?

**Authors: We had included Figure 3 in the revised version.**

DISCUSSION
L383, long ‘history’

**Authors: Corrected**

ACKNOWLEDGEMENTS
Manuscript also improved by suggestions by reviewers.
**Authors: Done**

SUPPORTING INFORMATION
Much better.

**Authors: Thank you.**

Reviewer #3: First of all, I would like to congratulate the authors for their impresive work in taking into account the large number of comments that were made in the previous version of the manuscript.

**Authors: Thank you for your support and for your previous suggestions.**

I have still two major issues with the manuscript as it now stands:

- Hypothesis: Hypothesis 1 is very long and rather hard to follow. I would recommend to split it up, for instance into a) functional richness, evenness and divergence of resource use traits are expected to decline with …., b) low matrix quality is expected to lead to stronger decline in …., c) trait distribution is expected to become more skewed towards …, d) functional richness, evenness and divergence of dispersal trais are expected to … I would switch hypothesis 2 and 1, hypothesis 2 present how you expect fragmentation effects operating at different scale to interact with each other. Hypothesis 2 is therefore more general. In hypothesis 2 you write: „distance to fragment edge tend to increase in small … fragments“, how is that possible? In smaller fragments every single points should be closer to the edge than in larger fragments.

**Authors: Thank you for the suggestion. We have split the presentation of the hypothesis accordingly. We have also clarified and replaced “distance to fragment edge tend to decrease in small…fragments”.**

- Discussion: Fragmentation and especially reduction in habitat area is a process that is usually expected to lead to declining biodiversity, yet looking at Figure 2 it seems that this is not the case here. Based on this main results I am missing a more explicit discussion of the absence of negative direct and indirect effect of reduced fragment area on functional diversity. This sounds rather provocative but your results tend to show that smaller forest patches do not have lower functional richness and evenness compared to larger patches but they even have higher functional divergence. I think that such discussion would be particularly interesting around the lines 368-369, where the opposite is expected. I think that this manuscript would be greatly enhanced by further interpretation of these results and their potential implications.

**Authors: Larger fragments (area?) tended to include more interior plots (>200 m from the forest edge), whereas smaller fragments (i.e. < 20 ha) generally included far fewer interior plots due to their smaller size. The observed decrease in functional divergence may be explained by the fact that interior plots showed higher functional divergence than plots located closer to the edge. The decrease in functional divergence might have not been so significant in smaller fragments because most studied plots were found within 100m from the forest edges. We have clarified this in the Discussion (494-497).**

Minor comments:

- line 230: replace „determine“, maybe use „measure“ instead

**Authors: Corrected**

- line 259: which package was used to compute the SEMs?

**Authors: Corrected**

- line 309-312: does the decrease of CWM for shade tolerance with distance to forest edge means that plants are more shade-tolerant closer to the edge? This sounds rather counter-intuitive, do you have some explanation for this?

**Authors: Shade tolerance significantly declined with increasing distance from the plot to forest edge (see Table 1), while we found an increase in light-demanding species characteristic of edge habitats (i.e. pioneers and light-demanding non-pioneers).**
